# In Situ Investigation of Grain Evolution of 300M Steel in Isothermal Holding Process

**DOI:** 10.3390/ma11101862

**Published:** 2018-09-30

**Authors:** Rongchuang Chen, Zhizhen Zheng, Jianjun Li, Ning Li, Fei Feng

**Affiliations:** School of Materials Science and Engineering, Huazhong University of Science and Technology, and State Key Laboratory of Materials Processing and Die & Mould Technology, Huazhong 430074, China; crc@hust.edu.cn (R.C.); jianjun@mail.hust.edu.cn (J.L.); hslining@mail.hust.edu.cn (N.L.); fengfei@hust.edu.cn (F.F.)

**Keywords:** grain size evolution, grain growth, in situ observation, 300M steel

## Abstract

The relationships between initial microstructures, process parameters, and grain evolutions in isothermal holdings have drawn wide attention in recent years, but the grain growth behaviors of 300M steel were not well understood, resulting in a failure in precise microstructure controlling in heat treatment. In this work, in situ observations were carried out to characterize the grain evolutions of 300M steel with varying holding time, holding temperatures, and initial microstructures. The intriguing finding was that the grain refinement by austenization of 300M steel was followed by a dramatic grain growth in the initial stage of holding (≤~600 s), and with increasing time (~600–7200 s), the average grain size appeared to have a limit value at specific temperatures. The austenization process accelerated the grain growth by generating large quantity of grain boundaries at the initial stage of holdings, and the growth rate gradually slowed down after holding for ~600 s because the driven force was weakened due to the reduction of grain boundary energy. The initial structure and the initial grain size of 300M steel had no obvious influences on the grain size evolutions. The mechanisms of grain growth were analyzed based on in situ observations and transmission electron microscope (TEM) characterizations. A grain evolution model considering the grain boundary migration of 300M steel was established for the isothermal holding process. Good agreement was obtained between the in situ observation results and the model calculation results. This investigation aimed to understand fundamentally the grain evolutions of 300M steel in the isothermal holding process.

## 1. Introduction

The 300M steel is extensively used in manufacturing of large structural parts of aircraft landing gear and nuclear power plants seal head due to its excellent mechanical properties (σ_b_ ≥ 1800 MPa) [1]. These parts are usually heat-treated to meet specific mechanical performance requirements. However, the grain evolutions of 300M steel in isothermal holdings were not well understood, resulting in a failure in precise microstructure controlling, and therefore a reduction in the service performance of these parts.

In isothermal holdings of steels, austenite nucleated and grew at pearlite colonies and at ferrite phase boundaries. Then, grains could either be refined or coarsened depending on the holding time and temperatures [2]. It was reported by Karthikeyan et al. [3] that the grain size of 9Cr-1Mo steel was refined from 26 to 12 μm via high temperature holdings. The grain size of 22MnB5 carbon steel was significantly refined by short austenization at 900 °C for 2 s, and the tensile properties and hardness were greatly enhanced [4], whereas, high temperature holdings for a long period of time could lead to grain coarsening of steels. Fernández et al. [5] carried out research on grain size evolution of 16MnNi4 steel in isothermal holdings via metallography, and it showed that the dramatic growth of grains was followed by a slow growth during holding at 1050–300 °C. The result of Schino et al [6] by metallographic method showed that the austenite grains of a low nickel steel grew dramatically within the holding time of 0–5 min, but grains changed extremely slowly after ~5 min holding. The grain growth of a Cr–Mo–V Steel was investigated by Li et al. [7] via interrupted quenching, and it showed that the austenite grains had grown dramatically after several hours holding. It was generally believed that in the holding time, holding temperature and initial microstructures had influences on the grain growth of steels. Xu et al. [8] reported the initial grain size had influence on grain growth of a dual phase steel in isothermal holding, and the time exponent in the grain growth model was temperature dependent. Humphreys et al. [9] pointed out that the grain growth of steels was accomplished by grain boundary migration under the driven force of grain boundary curvature. Specifically, for 300M steel, the influences of holding time (5–120 min) and temperatures (850–1050 °C) on grain growth were investigated by Zhang et al. [10] via interrupted quenching, and several other investigations [1,11] on the grain growth were carried out as well. But the influences of initial grain size and initial structure were unclear, and a detailed description of the grain evolutions of 300M steel in isothermal holdings has not been established.

In situ methods allowed direct observations of grain size variations without altering the test conditions, and they were successfully used in the characterization of microstructure evolutions of steels for the past few years. Bulk methods such as ultrasonic techniques [12,13] and the differential scanning calorimeter method [14,15] achieved fast and in situ grain size determinations with a few experiments, but the grain growths were not directly observed, and they should not be used independent of metallographic observations [16]. As a surface characterization method, the in situ observations [17] have gained much attention due to its directness, and it has been adopted in the researches of solidification [18], phase transformation [16], and the grain growth [1] of steels recently. However, in situ investigations on grain evolutions of 300M steel in isothermal holding process are still lacking, and a systematic research on grain evolutions of 300M steel based on in situ experiments is still in urgent need.

In the present investigation, the influences of holding temperatures, holding time, initial structures, and initial grain sizes on grain evolutions of 300M steel will be investigated by in situ observations. The mechanisms of grain growth will be discussed. Finally, the grain size evolution model of 300M steel in the isothermal holding process will be established.

## 2. Materials and Experiments

### 2.1. Materials

**Figure 1:**
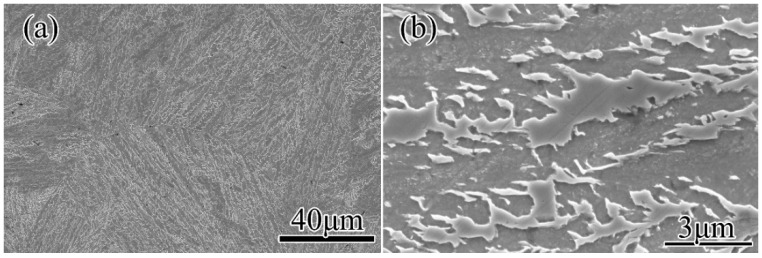
Microstructures of as-received material.

The as received material was annealed, and the initial microstructures were obtained via etching. The initial structure was bainite (Figure 1) and the initial grain size was 37.7 μm. The chemical compositions are shown in Table 1.

### 2.2. Obtaining of Different Initial Microstructures

**Figure 2:**
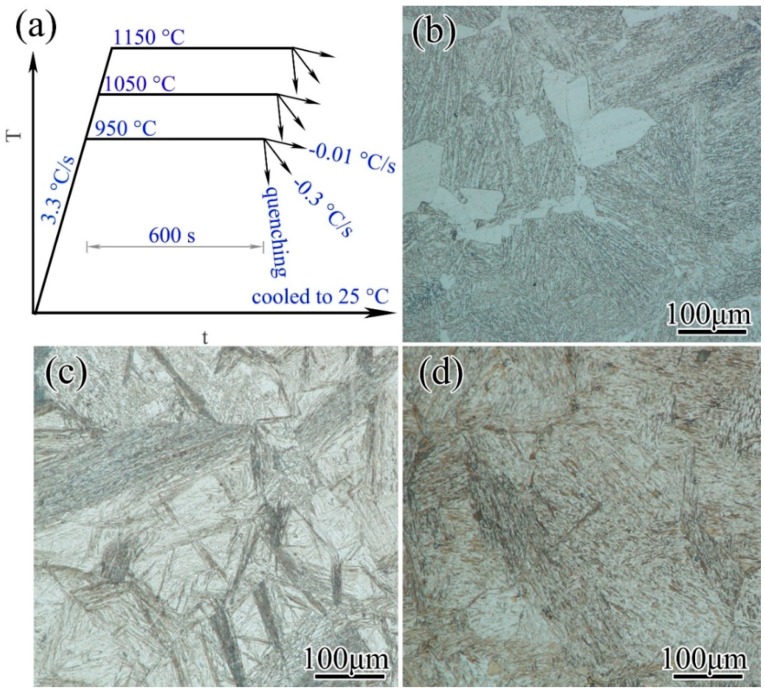
Temperature curves and microstructures of 300M steel. The temperature curves were shown in (**a**). It showed the etching results of specimens held at 1150 °C for 600 s and cooled at (**b**) −0.01 °C/s, (**c**) −0.3 °C/s, and by (**d**) quenching. The structures were (b) pearlite + ferrite, (c) bainite + martensite, and (d) martensite, respectively.

Specimens with various grain sizes could be obtained via holding at various temperatures (950, 1050, and 1150 °C) for a specified length of time (600 s). Different phases could be obtained via cooling at various rates (−0.01, −0.3 °C/s, and quenching) [16]. To obtain the initial microstructures with different grain sizes and different structures, the as-received materials were cut into cubes of 50 × 50 × 50 mm^3^ and heat-treated in an environmental furnace (SRJX-10-13, Subo Instrument Co., Yangzhou, China) according to the time-temperatures curves illustrated in Figure 2. Hence, nine groups of specimens with various grain sizes and structures could be obtained. These specimens were used in the subsequent heat-treatments to investigate the influences of initial grain sizes and structures. The grain sizes obtained via holding were 29, 57, and 90 μm, respectively.

### 2.3. In Situ Observation Tests

**Figure 3:**
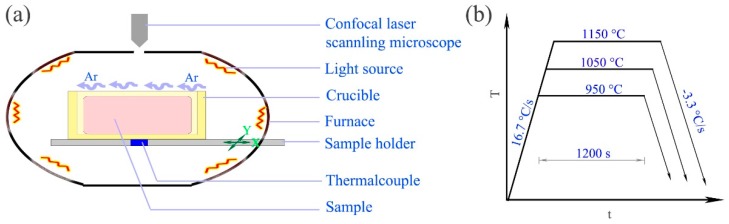
Illustration of the experimental setup and temperature curves of the in situ observation experiments: (**a**) illustration of the experimental setup; (**b**) temperature curves of the in situ observation experiments.

In situ observations were carried out on a high-temperature confocal laser scanning microscope (VL2000DX-SVF17SP, Yonekura MFG Co., Fukuoka, Japan), as shown in Figure 3a. The steel samples were placed in a crucible, whose temperatures were measured by a thermalcouple. The temperatures were adjusted continuously via the light sources controlled by a computer. The microstructure evolutions could be observed on the upper surfaces of specimens by a confocal laser scanning microscope (CLSM) due to the alloy elements’ volatilization at grain boundaries. The technical details of the high temperature confocal laser scanning microscope (HTCLSM) could be referred to the literatures [16,17]. Specimens were 2.5 mm of radius and 2 mm of height. The specimens observed surfaces were mechanically polished. The specimens were heated at a unified heating rate (16.7 °C/s) to holding temperatures. The temperature curves in the in situ observation experiments were shown in Figure 3b. Samples were heated to 950, 1050, and 1150 °C at 16.7 °C/s, isothermal held for 1200 s, and cooled at −3.3 °C/s to 25 °C. An argon flow of ~200 mL/min was used in the tests.

### 2.4. Characterizations on Optical Microscope and Transmission Electron Microscope

The cooled specimens in in situ experiments were mechanically polished and etched using etchant containing 22% detergent, 56% saturated picric acid, 0.17% hydrochloric acid, and balanced carbon tetrachloride (volume percentage). The microstructure photos were then taken on an optical microscope (VHX-1000C, Keyence Co., Osaka, Japan). Grain sizes were determined according to the linear intercept method in ASTM 112-13. The specimens for the transmission electron microscope (TEM, TECNAI G2 F30, FEI Co., Hillsboro, OR, America) tests were heated to 900 °C at 16.7 °C/s, held for 5 s, and water quenched. The TEM characterizations were carried out according to a standard procedure [16].

## 3. Results

### 3.1. In Situ Observations of Grain Evolutions

**Figure 4:**
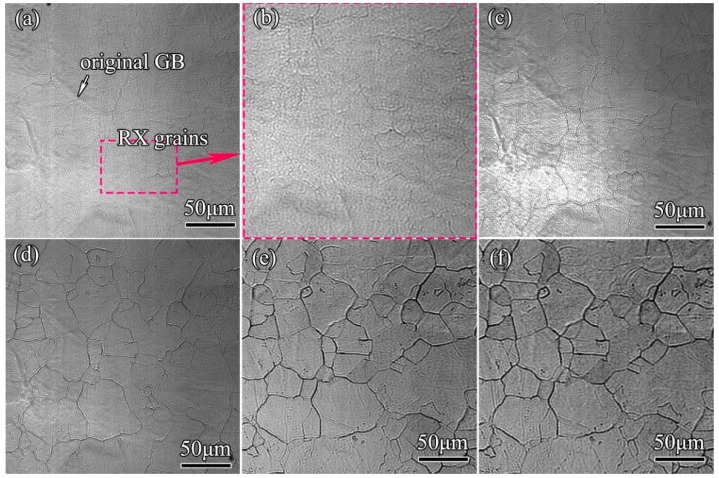
In situ observation results showing the microstructure evolutions of 300M steel heated at 16.7 °C/s to 1000 °C, and then isothermal held for (**a**) 240 s; (**c**) 266 s; (**d**) 316 s; (**e**) 523 s; and (**f**) 713 s. The zoomed view of (**a**) was shown in (**b**). The “GB” and “RX” denote “grain boundary” and “recrystallized”, respectively.

The grain evolutions of 300M steel in the isothermal holdings are shown in Figure 4. The microstructures underwent austenization and grain growth sequentially in holdings, and as shown in Figure 4a many small austenite grains (~14 μm) emerged in initial coarse grains, resulting in a significant grain refinement compared with initial grain size (38 μm) at 25 °C by metallography. As austenization was completed, small austenite grains grew by grain boundary discontinuous migrations and grain consumptions by surrounding grains (Figure 4b) [19,20]. With the further increase of the holding time, the grain growth gradually slowed down, and the grains underwent only slight change (Figure 4c–e).

The grain evolutions of steels in a relative short time holding (0–~10 min) were less revealed, because very complicated microstructure evolutions occurred in this process; for example, austenization, microstructure homogenization, and cementite dissolution, many of which had combined effects on austenite grain evolutions. The results in Figure 4 showed that the grains were refined (~14 μm) due to austenization in the early stage of holding, and grain coarsened (45 μm) thereafter, which was different from what was generally expected, that the grains would definitely grow coarser. It could be explained by the in situ observations that the grain refinement was due to austenization, but further observations ought to be carried out for a detailed description of the grain evolutions of 300M steel, and the influences of initial microstructures and heat-treatment parameters on grain evolutions should be investigated.

### 3.2. Influence of Holding Temperature and Time

**Figure 5:**
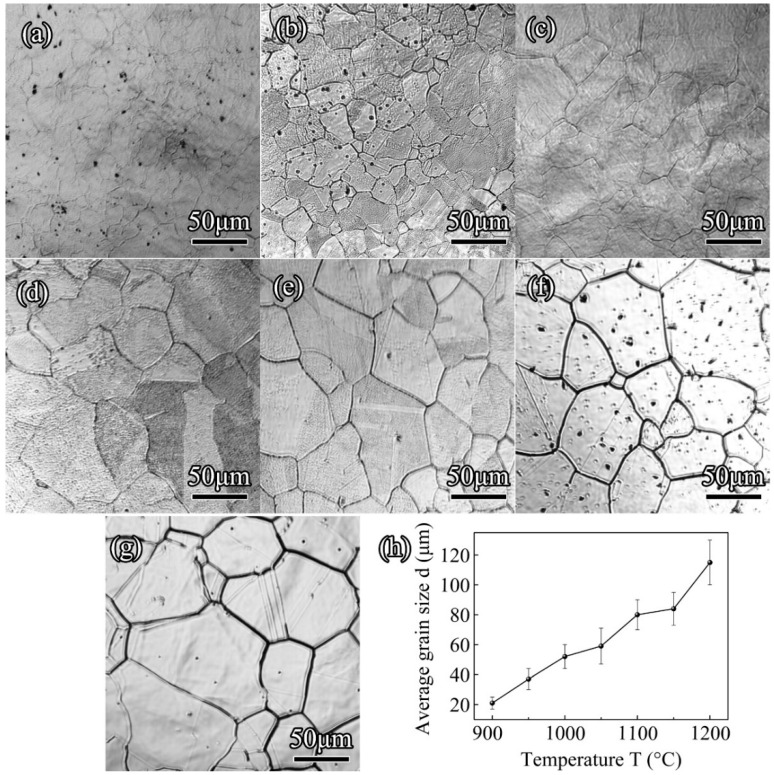
In situ observation results of 300M steel after heating at 16.7 °C/s to (**a**) 900 °C, (**b**) 950 °C, (**c**) 1000 °C; (**d**) 1050 °C; (**e**) 1100 °C; (**f**) 1150 °C; (**g**) 1200 °C, and isothermal held for 1200 s; (**g**) showed the grain sizes with increasing holding temperatures.

The microstructure evolutions of 300M steel with varying holding temperatures were shown in Figure 5. After holding at a relative low holding temperature of 900 °C (Figure 5a) for 1200 s, fully austenitized, equiaxed, and small grains with an average size of 21 μm were formed. The average grain sizes increased from 37, 52, to 59 μm while the holding temperatures increased from 950, 1000, to 1050 °C, as shown in Figure 5b–d. The grain boundaries were obviously straightened at the holding temperature of 1100, 1150, and 1200 °C in Figure 5e–g, and grain coarsening took place in the material, indicating that the grain growth was driven by decreasing grain boundary energy. The relationships between the holding temperatures and the average grain sizes are shown in Figure 5h. The average grain sizes gradually increased from 21 to 115 μm when the holding temperatures increased from 900 to 1200 °C. It is worth noting that the dots showing on the surface of the specimen in Figure 5a,b,f, as mentioned in other literatures [21,22], might occur under insufficient argon flow.

**Figure 6:**
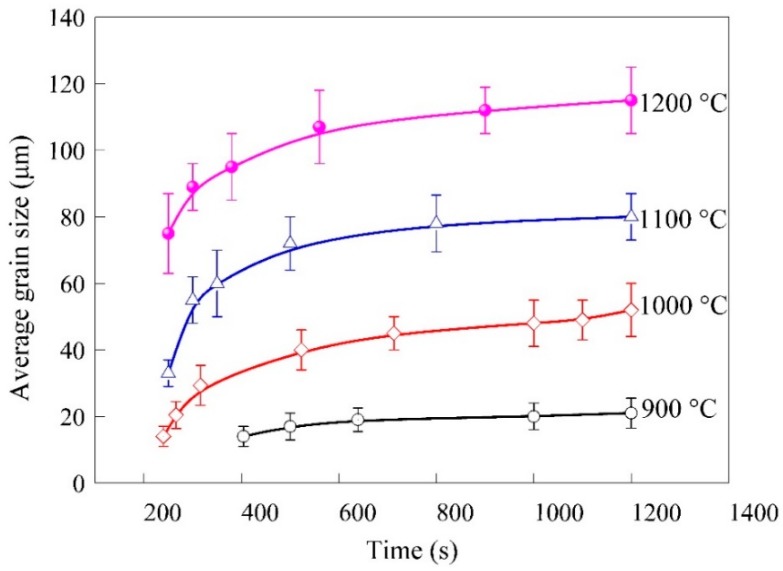
The grain size evolutions during holding for 1200 s.

The average grain size evolutions of 300M steel during isothermal holdings are shown in Figure 6. The grain morphologies were invisible within the holding time of ~200 s in all test conditions mainly because the austenization was in progress after a quick heating, and enough heat accumulation was needed for the evaporation of alloying elements. The average grain sizes increased with holding time, and the grains grew drastically in the initial stage. It could be explained that the interfaces generated during phase transitions accelerated the grain growth of austenite, and as shown in literature [9,22] that the grain growth of austenite was driven by grain boundary curvature. But the growth rates gradually slowed down after holding for ~600 s because the driving force was weakened due to the reduction of grain boundary energy. The grains grew extremely slowly after holding for 1200 s. Moreover, the grain boundary traces were left on the specimen surfaces due to evaporation of alloying elements, and some traces would not disappear once generated, which made subsequent observations difficult [1,16]. Therefore, ex situ and in situ results should be combined to make an accurate description of grain growth behaviour of 300M steel.

### 3.3. Influence of Initial Microstructures

**Figure 7:**
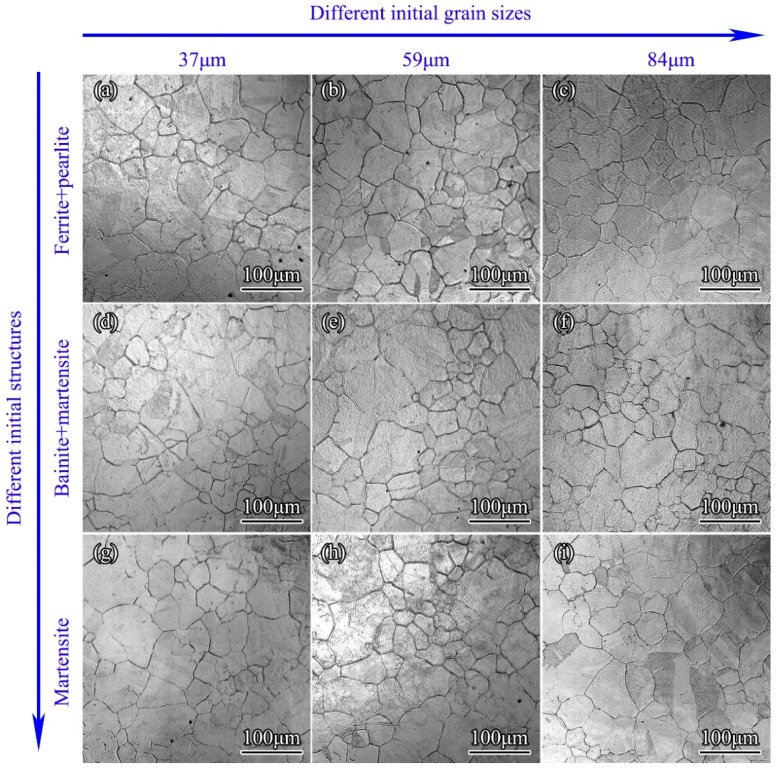
In situ observation results of 300M steel with different initial microstructures after holding at 1050 °C for 1200 s. The initial grain sizes and initial structures for each experiment were marked on the edge. The average grain sizes were 58, 55, 54, 56, 63, 55, 53, 58 and 65 μm, respectively.

The influences of the initial structures (martensite, bainite, and pearlite) and the initial grain size were examined experimentally. Specimens were heated to different temperatures (950, 1050, and 1150 °C), held for 600 s, and cooled at different cooling rates to obtain specimens with different initial microstructures. Then these specimens were reheated to 1050 °C. The microstructures after reheatings and holdings for 1200 s were shown in Figure 7. Both the initial grain sizes and initial structures showed no obvious influences on grain growth, and all grain sizes dropped within the range of 59 ± 6 μm, indicating that the average grain sizes were independent of initial microstructures of the material at room temperature. The evaporation of alloying elements at prior austenite grain boundaries meant boundary segregation. From this point of view, there should be some difference in behaviors of austenite created from various input microstructures, but the results in Figure 7 showed that the initial grain sizes and initial structures had no influence on grain growth of 300M steel in isothermal holdings. It could be explained that the element segregation in the grain boundaries only occurred in very small local areas, and the diffusion of the elements in the phase transition “stir” the elements in local areas, resulting in an elimination of the influences of element segregation. Besides, for steels which underwent phase transitions during holdings, strains would be introduced in the material due to phase transition, and full recrystallization would occur, resulting in a grain refinement during heating and holding. Thus, the grain sizes after recrystallization were determined by the holding temperatures and time, rather than by the microstructures at room temperatures.

### 3.4. Comparison with the Metallographic Results

**Figure 8:**
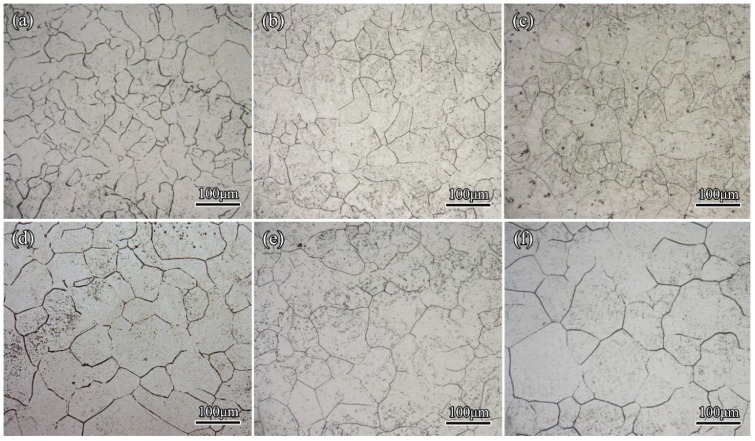
The ex-situ observed microstructures of 300M steel after holdings at (**a**) 900 °C; (**b**) 950 °C; (**c**) 1000 °C; (**d**) 1050 °C; (**e**) 1100 °C; and (**f**) 1150 °C for 1200 s. The average grain sizes were 28, 42, 46, 66, 73, and 81 μm, respectively.

Since the traditional metallographic characterizations were proved effective in most cases, a comparison between the metallographic results and the in situ observations was undertaken. The microstructures by metallography were shown in Figure 8. The average grain sizes of 300M steel increased with holding temperatures, and this was consistent with the in situ results.

**Figure 9:**
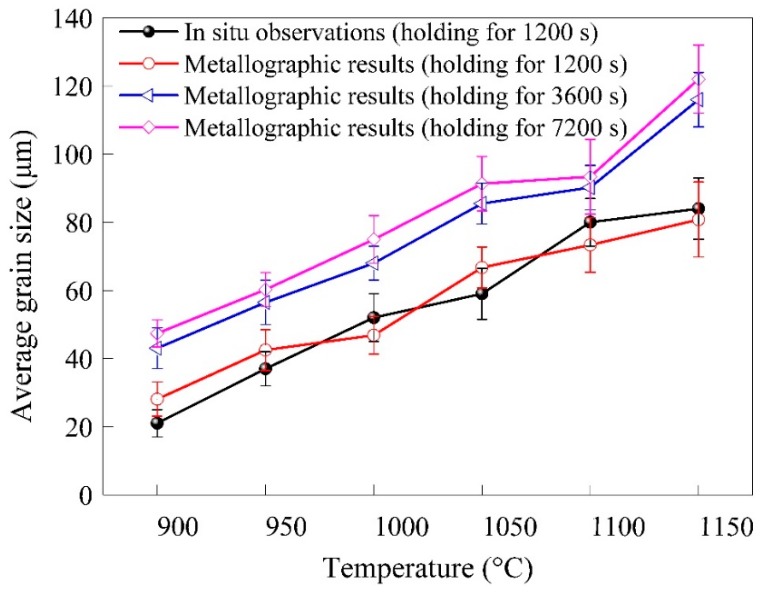
Comparison of the metallographic results and the in situ results.

Ex situ experiments were carried out to investigate the grain growth behaviors of 300M steel in long time holdings (~7200 s). The average grain sizes after holdings at various temperatures for 3600 and 7200 s are also shown in Figure 9. A moderate grain growth was found between 1200 and 3600 s, but a very slight grain growth was found between the holding time of 3600 and 7200 s according to metallographic results. It could be explained that as the grain boundaries were gradually straightened, the driving force of grain boundary migrations and grain growth was weakened. The comparison of the metallographic results and the in-situ results in Figure 9 showed that the average grain sizes in ex situ experiments agreed well with that of in situ results.

## 4. Discussions

### 4.1. Mechanisms of Grain Refinement and Grain Growth

**Figure 10:**
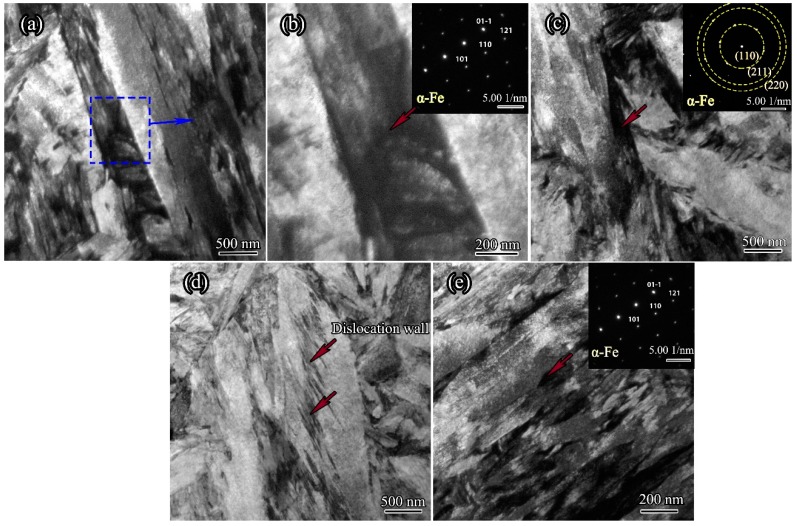
Bright field image showing the microstructures of 300M steel obtained by isothermal holding at 900 °C for 5 s and quenching: (**a**) ferrite lath in martensite; (**b**) the zoomed view of ferrite lath; (**c**) distorted ferrite due to martensite transformation; (**d**) dislocation wall within a martensite lath; and (**e**) initial formed nuclei on the interfaces of the martensitic laths.

It was revealed by in situ observations that the 300M steel underwent full recrystallization during holdings, and the reason for the recrystallization was the strains and dislocations which were introduced by phase transitions, but the strains and dislocations could not be observed on the HTCLSM due to a relative low magnification. Thus, the TEM characterizations were carried out. The specimens were heated to 900 °C at 16.7 °C/s, held for 5 s, and water quenched. The bright field image in Figure 10 shows that martensite laths including ferrite and cementite were formed (Figure 10a,b), and the ferrite lath was distorted due to martensite transformation (Figure 10c). It is also shown in Figure 10d that a dislocation wall was formed within martensite laths. Chen et al. [20] reported that the strain and dislocations could cause recrystallization of 300M steel at high temperatures. The martensite laths which contained large strains and high dislocation densities could act as prior nucleation sites for recrystallization during reheating. In austenization, the austenite nuclei sites were possible to maintain using an interrupted quenching technique according to Shtansky et al. [23]. Accordingly, the initial formed nuclei in holdings of 300M steel were observed, as shown in Figure 10e that the nuclei were formed on the interfaces of the martensitic laths. It could be explained that austenite transformations were more easily to take place at phase interfaces due to a higher dislocation density and a higher alloying elements content.

**Figure 11:**
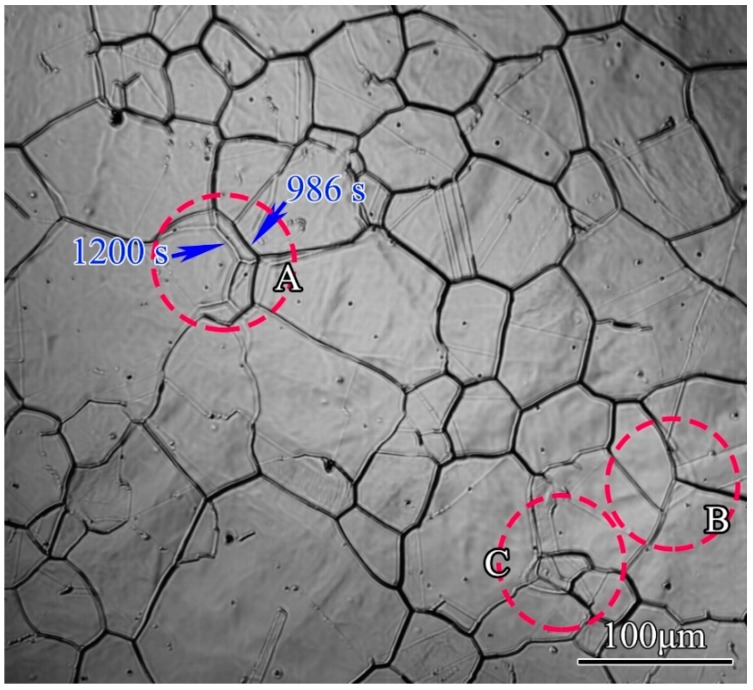
Grain boundary migration at 1200 °C.

The grain boundary traces were generated due to evaporation of alloying elements on the specimen surfaces, and some would not disappear thereafter. Thus, it was possible to distinguish the grain boundary migrations via the traces. The grain boundary migrations at the holding temperature of 1200 °C are shown in Figure 11. It could be seen that the convex parts of grains were flattened by grain boundary migrations in circle A and B, and a small grain was consumed by surrounding grains by grain boundary migrations in circle C, resulting in an increase of average grain size. The grain boundary migrations were the main mechanisms for grain growth. But the models that were established by Chen et al. [1], Zhang et al. [10], and Luo et al. [11], had not considered the grain boundary migrations, and a physical based grain growth model based on quantitative analysis of in situ results was still in need.

### 4.2. Modelling of Grain Size Evolutions

The driving force of grain boundary migrations was the grain boundary curvatures, and the driving pressure (*P*) could be calculated based on Equation (1) and expressed as [24]:

(1) $$P=\frac{{\alpha \gamma }_{b}}{\bar{R}}$$

where the *α* was the geometric constant, $\gamma_{b}$ the grain boundary energy, and $\bar{R}$ the curvature radius. The velocity of grain boundary migration (*v*) was proportional to the driving pressure [25]:

(2) $$v=\frac{d\bar{R}}{dt}=c_{1}P$$

where $c_{1}$ was a material constant, and $\bar{R}$ was the average grain radius. It was argued by Grey et al. [24] that there was a limit size of grain growth, and it was also found in this investigation that the grain growth almost disappeared after holdings for 7200 s. Accordingly, the velocity could be calculated based on Equation (2) and expressed as:

(3) $$v=\frac{d\bar{R}}{dt}=c_{1}(P+C)$$

where *C* was a term considering the limiting size of phase change material. Combining Equations (1) and (3), the following could be obtained [24]:

(4) $$v=\frac{d\bar{R}}{dt}=c_{1}\left(\frac{{\alpha \gamma }_{b}}{\bar{R}}+C\right)$$

**Figure 12:**
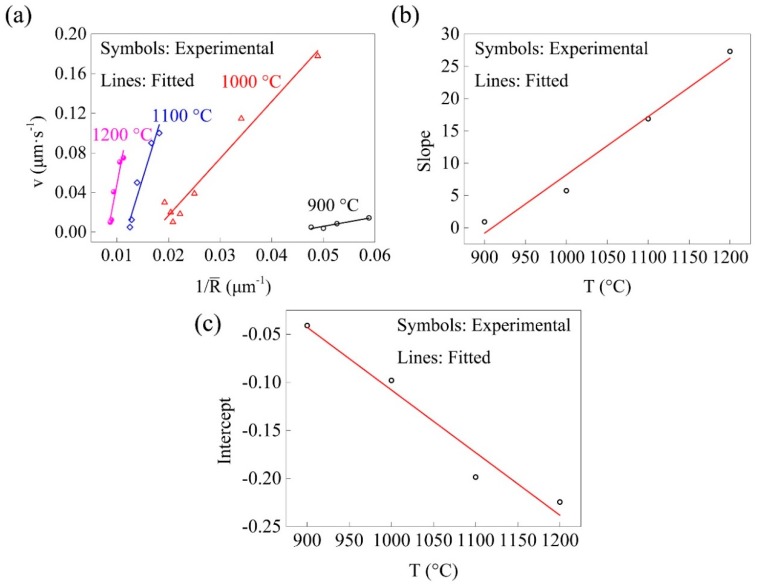
Calculation procedure to obtain the model parameters. It shows (**a**) the relationship between *v* and $1/\bar{R}$; (**b**) the slope of the fitted lines of *v* and $1/\bar{R}$; and (**c**) the intercept of the fitting lines of *v* and $1/\bar{R}$.

In Equation (4), the relationship between the grain boundary migration velocity (*v*) and the average grain radius ($1/\bar{R}$) could be obtained via in situ observations, shown in Figure 12a. An approximate linear relationship could be found between the grain boundary migration velocity (*v*) and the average grain radius ($1/\bar{R}$), and the slope and the intercept also followed an approximate liner relationship. In a more general form, the relationships of the average grain radius, the holding temperature, and the holding time could be expressed as:

(5) $$\frac{d\bar{R}}{dt}=\frac{m_{1}{T+m}_{2}}{\bar{R}}+m_{3}T+m_{4}$$

where $m_{1}$ and $m_{2}$ were the slopes of the fitting lines in Figure 12b,c, and $m_{3}$ and $m_{4}$ were the intercepts. The grain morphologies were invisible within the holding time of ~200 s in all holdings according to the in situ results, and the grain sizes that could be initially visualized was extremely small. Assuming that the average grain size at *t_s_* was *R_0_*, the relationships of the average grain radius, the holding temperature, and the holding time could be calculated by integrating both sides of Equation (5) against time (*t*), applying the initial value ($\bar{R}{|}_{t=t_{s}}=R_{0}$), and reorder the constants as $k_{1}$–$k_{6}$. Thus, the following expression was obtained:

(6) $$\bar{R}=\left(k_{1}T+k_{2}\right)\ln\left(t\right)+\left(k_{3}T+k_{4}\right)t+k_{5}T+k_{6}$$

A multiple non-linear regression could be applied to obtain the $k_{1}$–$k_{6}$ in Equation (6). The average grain size ($\bar{d}$) was twice the radius ($\bar{R}$) and expressed as follows:

(7) $$\bar{d}=\left(-0.05618T+106.4\right)\ln\left(t\right)+\left(9.557\times 10^{-5}T-0.1464\right)t+0.5988T-843.6$$

**Figure 13:**
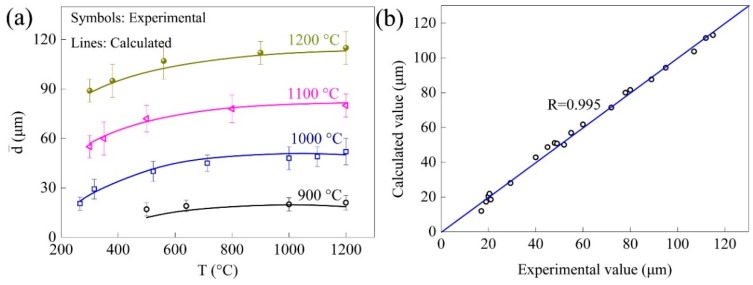
Comparison of the calculated and experimental grain size. It showed (**a**) comparison of calculated and experimental grain size; and (**b**) a scatter plot drawn from calculated and experimental grain size.

The comparison of the calculated and experimental grain sizes according to Equation (7) is shown in Figure 13a, and a scatter plot drawn from calculated and experimental grain size is shown in Figure 13b. The mean relative error was calculated to be 5.36%, and the R value for evaluating the goodness of fitting was 0.995, indicating that the model was precise. For comparison, the Burke and Turnbull model [24] was also implemented, and the mean relative error was calculated to be 11.2%, showing that the model established in this investigation was more accurate.

### 4.3. Industrial Implications

The grain size evolution of 300M steel is a major concern in the high temperature holding process, and precise grain size controlling is of paramount importance in heat treatment of heavy forgings, for example, aeroplane landing gears. In engineering practice, the holding time of heavy forgings would be as long as several hours because it needed enough time for heat transfer, and too fast a heating or cooling rate will cause cracks in the parts. Thus, the average grain sizes were largely determined by the holding temperatures. It was revealed in this investigation that the recrystallization occurred in heating due to austenization, and the coarse grains could be significantly refined. It meant that if the 300M steel heavy forgings were already coarse grained, additional deformation processes were not necessary for coarse grain elimination, and a holding at ~900 °C could solve the problem well. From another point of view, the controlling of temperature within a certain range was especially important to obtain fine grains for heavy forgings.

## 5. Conclusions

The grain evolutions of 300M steel in isothermal holding was investigated via in situ observations, metallography, and TEM observations. The following conclusions could be drawn:

(1) The microstructures of 300M steel underwent austenization and grain growth sequentially in holdings, and the small austenite grains grew by grain boundary discontinuous migrations and grain consumptions by surrounding grains. With the further increase of the holding time, the grain growth gradually slowed down, and the grains underwent only slight change.
(2) The average grain sizes gradually increased from 21 to 115 μm when the holding temperatures increased from 900 to 1200 °C. The grain size evolutions by in situ observations were different from the general expectations, and the grains could be refined at lower holding temperatures (~950 °C). It could be explained that the grain refinements were due to austenization.
(3) The initial grain sizes and initial structures had no influences on grain growth of 300M steel in isothermal holdings. The grain sizes after austenization of 300M steel were determined by the holding temperatures and time, rather than by the microstructures at room temperatures.
(4) It was shown by TEM results that the 300M steel underwent full recrystallization in the initial stage of holdings because of the strains and dislocations which were introduced by phase transitions.
(5) A grain growth model which has considered the grain boundary migrations based on quantitative analysis of in situ results was established. The mean relative error was 5.36%, showing an advantage in the grain size prediction precision over the Burke and Turnbull model.

## Figures and Tables

**Table 1:** Chemical compositions of the as-received 300M steel (weight %).

**Element**	C	Mn	V	Ni	Mo	Si	Cr	S	Fe
**Content**	0.39	0.808	0.086	1.824	0.435	2.562	0.896	0.017	Bal.
